# Protective roles of melatonin regulated mitochondrial DNA methylation and respiratory metabolism in ram sperm cryopreservation

**DOI:** 10.3389/fvets.2025.1657692

**Published:** 2025-09-03

**Authors:** Hai Hu, Liming Liu, Keqiang Wang, Jingjing Bao, Zengyuan Zhao, Rongzhen Zhong, Shangrong Xu, Yi Fang

**Affiliations:** ^1^Academy of Animal Science and Veterinary, Qinghai University, Xining, China; ^2^Inner Mongolia Shengle Biotechnology Co., Ltd., Hohhot, China; ^3^Key Laboratory of the Animal Production, Product Quality and Security, Ministry of Education, Jilin Agricultural University, Changchun, China; ^4^Hebei Tianhe Beef Cattle Breeding Co., Ltd., Shijiazhuang, China; ^5^Jilin Provincial Laboratory of Grassland Farming, State Key Laboratory of Black Soils Conservation and Utilization, Northeast Institute of Geography and Agroecology, Chinese Academy of Sciences, Changchun, China

**Keywords:** melatonin, methylation, mitochondrial DNA, sheep, sperm cryopreservation, respiratory chain complex

## Abstract

Mitochondrial dysfunction, especially compromised respiratory metabolism, is a serious obstacle of sperm cryopreservation. This study aims to determine the effect of melatonin supplement on respiratory metabolism of frozen-thawed ram sperm. Semen was slowly frozen with or without melatonin supplement, while fresh semen was used as a control. The results showed that melatonin clearly improved ATP production, oxygen consumption and respiratory chain complex activities, while it decreased reactive oxygen species and nitrite concentrations in frozen-thawed sperm (*p* < 0.05). Consequently, the viability, motility and fertility of frozen-thawed sperm were also recovered by melatonin. Strikingly, promoter methylation levels of several key mitochondrial respiratory chain genes were dramatically increased along with decreased expression levels in frozen-thawed sperm, which can be partially repaired by melatonin supplement (*p* < 0.05). This might be attributed to the expression change of *mtDNMT1* among three groups (*p* < 0.05). Furthermore, the declined expression of *MTNR1A* and *MTNR1B* were found in frozen-thawed sperm (*p* < 0.05). The treatment of melatonin receptor antagonist indicated that MTNR1A could have a key role in the regulation of melatonin on mitochondrial function of frozen-thawed sperm. Collectively, these findings provide a new perspective on the epigenetic regulation of sperm cryopreservation.

## 1 Introduction

As specialized mammalian cells, sperm retain abundant mitochondria accompanied by the disappearance of other organelles during maturation (1), emphasizing the importance of energy metabolism on sperm motility and fertility. In sperm cryopreservation, however, mitochondrial cryoinjury is one of the main causes of the low quality frozen-thawed sperm, given the important energy supply function and weak freezing tolerance of mitochondria (2). Frozen-thawed sperm have disruptions in metabolic enzymes and decreases in both ATP production and mitochondrial membrane potential (3, 4). Epigenetic modification of mitochondrial genes has been proved to influence sperm metabolism in various models (5–7). The changes of mitochondrial key enzyme activities could be determined by epigenetic modifications of several mitochondrial genes. Consequently, characterizing the epigenetic dynamics of mitochondrial genome may provide insights into frozen-thawed sperm dysmetabolism.

Sperm cryopreservation causes a series of abnormalities in epigenetic modifications, such as increased global DNA methylation level (8), reduced H3K9 acetylation and H3K4 methylation (9) and compromised DNA demethylation of paternal genome (10). Whereas most studies in sperm focused on nuclear genome DNA, epigenetic variation of mitochondrial DNA (mtDNA) has not been extensively studied. Although the local action of isoform 3 of DNMT1 as well as DNMT3A on mtDNA methylation patterns has been realized (11, 12), the precise roles of epigenetic modifications within mtDNA are unclear. Especially, the relationship between abnormal methylation and mitochondrial dysfunction still remains to be clarified in sperm.

Mitochondrion is both a primary synthesis site and principal target for melatonin (13), while melatonin can maintain the mitochondrial stability and increase ATP production (14). The ability of melatonin to promote animal reproductive functions has been well established (15, 16). Previous studies also indicate that melatonin protects sperm structure and function through its anti-oxidative and anti-apoptotic capacities (17, 18), which could also involve epigenetic modulation (19, 20). During sperm cryopreservation, melatonin can alleviate the inhibition of mitochondrial oxidative phosphorylation, not only by increasing the activity of respiratory chain complex, but also by promoting related gene expression (4). It implies that the effect of melatonin on sperm metabolism could involve the repair of enzymatic activity at the level of gene modification.

Hence, we investigated the effect of freezing-thawing and melatonin supplement on mitochondrial function and fertilizing ability of ram sperm. Furthermore, promoter methylation and expression levels of mitochondrial respiratory chain genes and *mtDNMT1* were detected to determine the potential mechanisms of melatonin and its receptors in mtDNA epigenetic regulation of frozen-thawed sperm.

## 2 Materials and methods

All procedures involving animals were approved by the Animal Ethics and Welfare Committee at the Academy of Animal Science and Veterinary, Qinghai University. All chemicals were purchased from Sigma Aldrich (St. Louis, United States), unless otherwise indicated.

### 2.1 Sample collection

The experiment was conducted during the physiological breeding season (late summer to late autumn). A total of 10 male small-tail Han sheep (2.5–3.0 years, 75–80 kg) had *ad libitum* access to water and to a 60:40 forage: concentrate ration as recommended by the National Research Council. Semen samples were collected using an artificial vagina containing water at 38–40 °C. Sperm concentration was calculated using a sperm density meter (SDM1, Minitube, Germany). Motility was estimated under 400× magnification using a phase-contrast microscope (BX60, Olympus, Japan). Ejaculates with volume of 0.7–2.0 mL, concentration >2.5 × 10^9^ sperm/mL and motility >80% were retained and pooled. Semen samples were pooled and then equally divided into 3 groups (fresh group, frozen group and melatonin group) to reduce the sample variance.

### 2.2 Sperm freezing and thawing

Basic semen extender (Tris-egg-yolk) was used to preserve fresh sperm (fresh group), containing an 80% solution of 3% glucose, 3% sodium citrate, 10 IU/mL penicillin and 10 IU/mL streptomycin in distilled water and 20% of egg yolk. Meanwhile, the freezing solution was 94% basic extender and 6% glycerol, supplemented with 0 or 10^−7^ M melatonin used in sperm cryopreservation (for frozen or melatonin group respectively).

Sperm cryopreservation was performed by slow freezing. Briefly, semen was diluted by 5-fold, chilled to 4 °C over a 2 h interval, then placed in 0.25 mL straws. Filled straws were placed 4 cm above liquid nitrogen for 7 min and then plunged into it. After 2 weeks, straws were thawed with a 40 °C water bath for 15 s. Survival sperm were selected by colloid single-layer centrifugation. Briefly, 1 mL thawed sample (~1 × 10^9^ sperm/mL) was gently placed on top of 2 mL 80% Percoll^™^ (GE Healthcare, Uppsala, Sweden) in PBS. The mixture was centrifuged at 600 g for 20 min at 25 °C. Then, the sperm pellets were washed with PBS and centrifuged again. The residue was transferred to a clean tube containing 1 mL diluent for density calculation and subsequent testing.

### 2.3 ATP production

The ATP production was detected with an ATP assay system bioluminescence detection kit (Promega, Madison, United States). After trypsinization, intracellular ATP was extracted from sperm in 100 μL 0.5% trichloroacetic acid. After neutralization and dilution by adding tris-acetate buffer (pH 7.75). Subsequently, absorbance at 570 nm was measured using a fluorescence microplate reader (Imark, Bio-Rad, United States). The ATP content was calculated based on a standard ATP curve.

### 2.4 Reactive oxygen species assay

Sperm intracellular reactive oxygen species (ROS) content was determined using a ROS assay kit (Beyotime, Shanghai, China). Pelleted samples were washed three times and then incubated with 10 μM 6-carboxy-2′-7′ dichlorodihydrofluorescein diacetate probe. After washing, the OD value was determined with a microplate reader at 488 and 525 nm. The ROS content was calculated using a standard curve.

### 2.5 Nitrite content

Sperm intracellular NO_2_^−^/NO_3_^−^ was determined using a total nitric oxide detection kit (Beyotime). The reaction was followed by the colorimetric detection of NO_2_^−^ as a colored azo dye, the product of Griess reaction that absorbs visible light at 540 nm. Following incubation, samples were washed with PBS and the OD value was determined with a microplate reader. The nitrite content was calculated using a standard curve.

### 2.6 Sperm kinematic evaluation

For detecting sperm viability, 5 μL of 1 mM propidium iodide (PI) was used to incubate for 5 min. After washing, samples testing was completed within 1 h using a flow cytometer (FACS Melody, Becton Dickinson, United States). Data for 20,000 cells per sample were stored in the list mode and analyzed with SuperCyt Analyst 3 software (Sierra Cytometry, Reno, United States).

Sperm progressive motility was determined using a computer-aided sperm analysis (CASA) system (Minitube, Tiefenbach, Germany). In brief, sperm concentration was diluted to ~2 × 10^7^ sperm/mL in PBS and incubated at 37 °C for 5 min. Then, 5 μL of sample was placed on a chamber-slide. For each sample, five non-consecutive microscopic fields were randomly chosen to examine under 400× magnification using a phase contrast microscope (Axio Scope A1, Zeiss, Germany).

### 2.7 *In vitro* fertilization

The *in vitro* fertilization (IVF) experiment was performed as described (21). Ovaries were collected and transported to the laboratory within 2 h. Cumulus-oocyte complexes (COCs) were released and transferred into maturation medium (20% heat-inactivated estrous sheep serum, 10 mg/L follicle stimulating hormone, 10 mg/L luteinizing hormone, 10 μg/L epidermal growth factor and 1 mg/L estradiol-17β in TCM199 medium) for a 24 h incubation at 39 °C with 5% CO_2_. After removing cumulus cells via hyaluronidase digestion, the mature oocytes were co-incubated with fresh or frozen-thawed sperm (~1 × 10^6^ sperm/mL) in synthetic oviduct fluid medium (20% estrous sheep serum and 10 mg/L heparin) for 22 h. Presumptive zygotes were washed to remove the sperm and transferred into a new synthetic oviduct fluid medium supplemented with 1% basal medium Eagle-essential amino acids, 1% modified Eagle medium-nonessential amino acids, 1 mM glutamine, and 6 g/L fatty acid-free bovine serum albumin. The cleavage and hatching rates were determined at 48 h and 7 days post-insemination, respectively. The hatching rate was calculated based on cleaved embryos.

### 2.8 Oxygen consumption

An extracellular flux analyzer (XF24, Seahorse Bioscience, United States) was used to detect sperm oxygen consumption. Briefly, sperm sample was adjusted to ~2 × 10^6^ sperm/mL and seeded on a plastic microplate coated with concanavalin A. After centrifuging at 1,200 × g for 2 min, 500 μL medium was added to each well. After 54 min, 1 μM FCCP or mT medium (control group) were added to corresponding wells. Furthermore, 1 μM antimycin A and 1 μM rotenone were added to each well after 78 min. The oxygen consumption rate (OCR) was normalized by the number of sperm present and reported as amol of O_2_ min^−1^ sperm^−1^. Mitochondrial basal and maximum OCR values were calculated by subtracting OCR values of FCCP group recorded at 54 and 78 min, respectively.

### 2.9 Respiratory chain complex activity

Activities of NADH-CoQ oxidoreductase (Complex I), CoQ-cytochrome C oxidoreductase (complex III), Cytochrome C oxidase (Complex IV) and ATP synthase (Complex V) were detected with corresponding activity assay kits (Solarbio, Beijing, China). The reaction was assessed by determining the decrease in absorbance with a diode-array spectrophotometer (Lambda 265, PerkinElmer, United States). The complex I, III and IV activities were calculated by measuring the declined rates at 340, 450 and 550 nm. The complex V activity was calculated by measuring the rate of Pi generation.

### 2.10 CpG methylation level of mtDNA

mtDNA was extracted according to a commercial protocol (G-Biosciences, St. Louis, United States). In brief, samples were gently homogenized in cold cell lysis buffer and then centrifuged at 700 g for 10 min to pellet the nucleus. The supernatant was centrifuged again at 12,000 g for 15 min to pellet mitochondria. The pellet was re-suspended in mitochondrial lysis buffer with proteinase K and incubated at 37 °C overnight. Thereafter, sample was extracted with isopropanol, rinsed with ethanol and dissolved in EDTA buffer.

DNA methylation levels of CpG sites in the promoter region of *ND1*, *ND3*, *CYTB*, *COX2*, *COX3* and *ATP6* were analyzed with EpiTYPER (MassARRAY system, Agena Biosciences, United States). Primers used for CpG methylation detection were listed in Table 1. The parameters for the thermal cycler were as follows: denaturation at 94 °C for 4 min; 45 cycles of 94 °C for 20 s, 56 °C for 30 s, 72 °C for 1 min; extension at 72 °C for 3 min. Then, products were electrophoresed using 2% agarose gel to confirm amplification. The CpG sites were unambiguously interrogated and genomic locations detailed. Mass spectra methylation ratios were generated using EpiTYPER, and unmethylated (0%) and methylated (100%) DNA samples were used as controls. DNA methylation content was measured by a methylated DNA quantification kit and calculated by averaging across a total of CpG sites.

**Table 1:** Primers used for CpG methylation detection.

Gene	Primer sequence	Product size (bp)	Coverage CpG sites
*ND1*	F: TTGTTAATTTATAAGGAGTGTTGTTATTT	382	7/11
	R: CCAACACAAAAATACACCCAAA		
*ND3*	F: TGAGTTTATTATAGTTTTATAGAAGGGAA	361	10/13
	R: TACTAAAAAAACATAAACCTCATCAAT		
*CYTB*	F: ATTAAAGATATTTGGGGTTTTTTTT	325	7/8
	R: ATTTCCTCAAAACTACTTTCCAACA		
*COX2*	F: GTTGGTAGAGAATTGGGTTTTTTTT	289	4/5
	R: ACCCATACAAAAACATCAATAAACCT		
*COX3*	F: TTATTTTGTTATATAGATGAGTTGGTTTT	355	7/7
	R: ACATCTAATTTACACCTAAAAAATTTCACA		
*ATP6*	F: GTAGTTGGAGGATTTGGGTGATAG	194	7/13
	R: CCCTAAAAAAACCCATTCATTCTAC		

### 2.11 RNA extraction and quantitative real-time PCR

Total RNA was extracted using TRIzol reagent from sperm samples. After removing genomic DNA, 1 μg total RNA was reverse transcribed into cDNA with first-strand cDNA synthesis mix with gDNA remover. A real-time PCR system (ViiA7, Applied Biosystems, United States) was used to quantify the relative abundance of specific transcripts. Relative mRNA expression levels were calculated using the 2^−ΔΔCt^ method with triplicate, and β-Actin was used as internal control. All primers used are listed in Table 2.

**Table 2:** Primers used for qRT-PCR analysis.

Gene	Primer sequence	T annealing (°C)	Size (bp)
*ND1*	F: TAACATTGTTGGTCCATACG	58	91
	R: ATGCTAGTGTGAGTGATAGG		
*ND3*	F: GAAGCCAGGTCACCTTTCAA	57	76
	R: TCCTGGAATCAACAAGCACA		
*CYTB*	F: CGGCTGACTAATCCGATACC	60	106
	R: TGGGAGTACATAGCCCATGA		
*COX2*	F: GGGAGAAGCCTTAGTAGAGATTCTC	60	77
	R: CGGGTGTCTACATCTAGGCCTACTGT		
*COX3*	F: CAGCCTAGTTCCTACCCACGAC	59	103
	R: CCCGTTGCTATGAAGAATGTTG		
*ATP6*	F: CGAACCTGAGCCCTAATA	59	84
	R: GTAGCTCCTCCGATTAGA		
*mtDNMT1*	F: TCCCTGGGCATGGCCGGCT	59	165
	R: CTCTTTCCAAATCTTGAGCCGC		
*MTNR1A*	F: GGAGGGTGAAACCTGACGAC	57	99
	R: CCCAGCAAATGGCAAAGAGG		
*MTNR1B*	F: GGCTCCGTCTTCAACATCACC	60	145
	R: GCAGAAGGACCAGCAGGGTG		
*β-Actin*	F: GTCATCACCATCGGCAATGA	57	182
	R: CGTGAATGCCGCAGGATT		

### 2.12 Statistical analysis

All data are expressed as the mean ± standard error from at least 3 independent biological replicates. Statistical analyses were performed with analysis of variance (ANOVA) and Duncan’s multiple range test. A Levene test was used to confirm homogeneity of variances. Differences were considered to be significant at *p* < 0.05.

## 3 Results

### 3.1 Effects of melatonin supplement on frozen-thawed ram sperm quality and fertility

**Figure 1:**
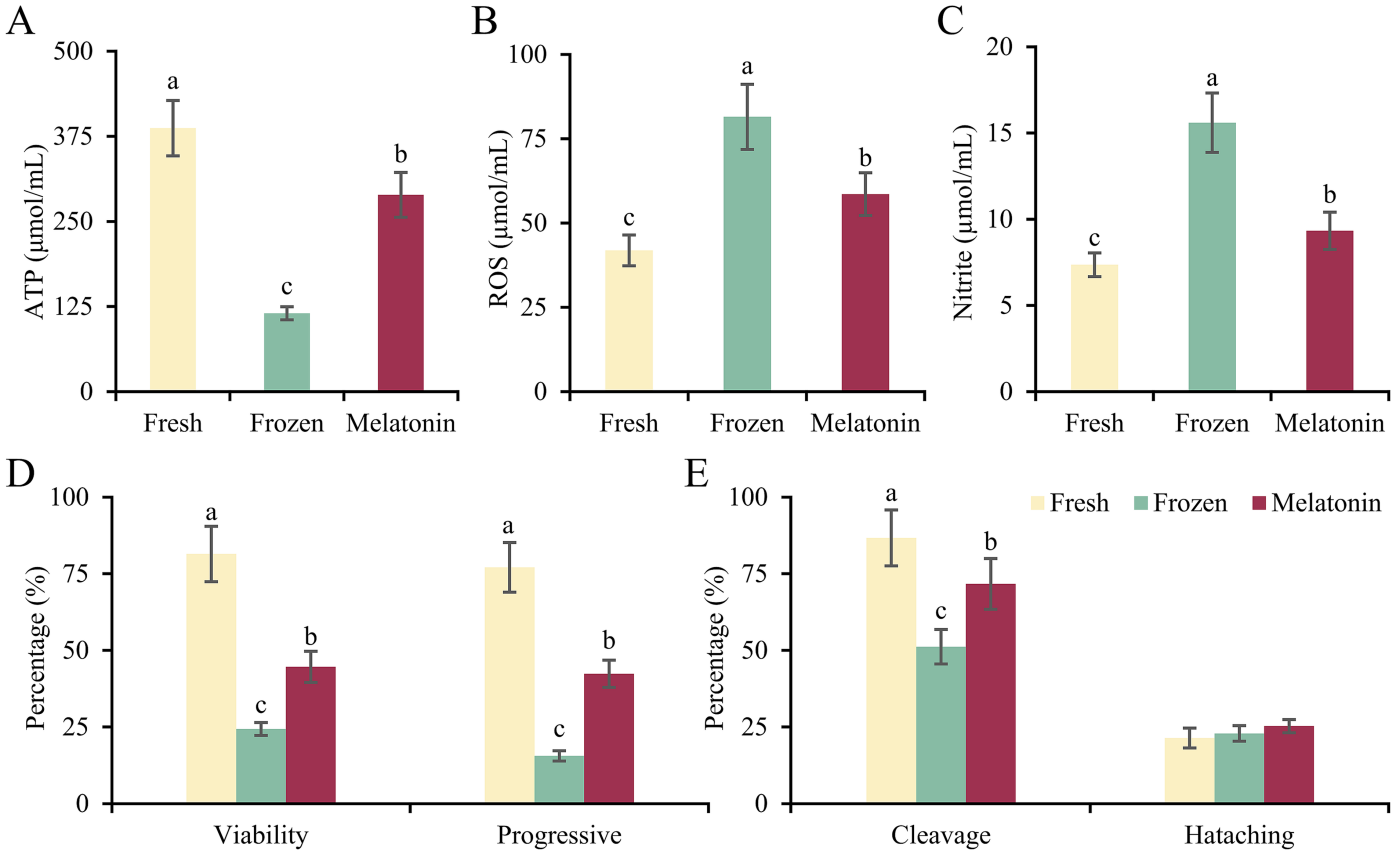
The parameters of sperm quality and fertility in fresh, frozen and melatonin groups. **(A–C)** The concentrations of ATP **(A)**, ROS **(B)** and nitrite **(C)** in the sperm from different groups. **(D)** The percentages of viable and progressive motile sperm in different groups. **(E)** The cleavage and hatching rates obtained from different groups in IVF experiment. Different letters indicate the significant difference between two groups with *p* < 0.05.

As shown in Figures 1A–C, freezing-thawing process led to decreased ATP content and increased ROS and nitrite accumulation in ram sperm (*p* < 0.05). The addition of melatonin in the freezing solution partially repaired these abnormal changes of frozen-thawed sperm, although it is inferior to fresh group (*p* < 0.05). The sperm kinematic analysis revealed that the ratios of total viable and progressively motile sperm in frozen group were just 29.90 and 20.23% of fresh group, while those of melatonin group could reach 54.80 and 54.99% of fresh group (*p* < 0.05, Figure 1D). Despite dramatically decreased cleavage rates obtained from frozen and melatonin groups in IVF experiment (*p* < 0.05), similar rates of hatched blastocysts were harvested from three groups (21.40, 22.90 and 25.30%) for fresh, frozen and melatonin groups, respectively (Figure 1E). It implies that the freezing injury in sperm would not cause the long-term effects on embryo survival.

### 3.2 Effects of melatonin supplement on frozen-thawed ram sperm respiratory chain complexes

**Figure 2:**
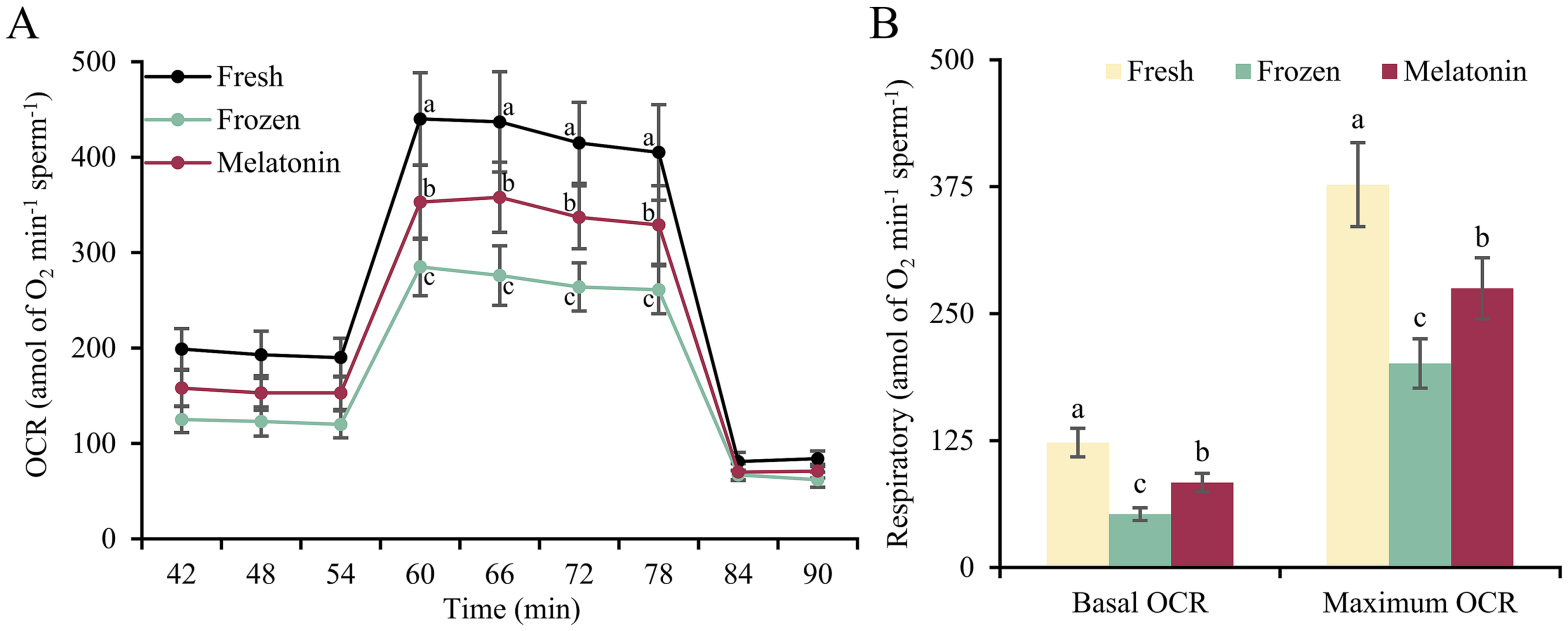
Sperm oxygen consumption in fresh, frozen and melatonin groups. **(A)** The curves for OCR in the sperm from different groups. **(B)** The basal and maximum OCR values in the sperm from different groups detected at 54 and 78 min, respectively. Different letters indicate the significant difference between two groups with *p* < 0.05.

The oxygen consumption detection showed that mitochondrial basal and maximum OCR in sperm were declined after freezing-thawing process (*p* < 0.05). However, melatonin supplement can promote aerobic respiration of frozen-thawed sperm remarkably (*p* < 0.05, Figure 2). Furthermore, this change was also supported by the activity detection of respiratory chain complexes. The activity of complex I, III, IV, and V showed a similar variation trend with 52.84%–67.00% decrease from fresh group to frozen group and 52.84%–102.15% increase from frozen group to melatonin group (Table 3).

**Table 3:** Sperm respiratory chain complex activities in fresh, frozen and melatonin groups.

Group	Complex I	Complex III	Complex IV	Complex V
Fresh	422.00 ± 41.30^a^	23.40 ± 3.01^a^	102.50 ± 11.4^a^	70.60 ± 7.74^a^
Frozen	199.00 ± 20.50^c^	8.27 ± 0.99^c^	47.50 ± 4.39^c^	23.30 ± 2.81^c^
Melatonin	338.00 ± 36.20^b^	15.10 ± 1.83^b^	72.60 ± 5.83^b^	47.10 ± 5.08^b^

Specific enzyme activity was expressed as U/mg prot. Within a column, different superscript letters indicate the significant difference between two groups with *p* < 0.05.

### 3.3 Effects of melatonin supplement on mtDNA methylation and gene expression in frozen-thawed ram sperm

**Figure 3:**
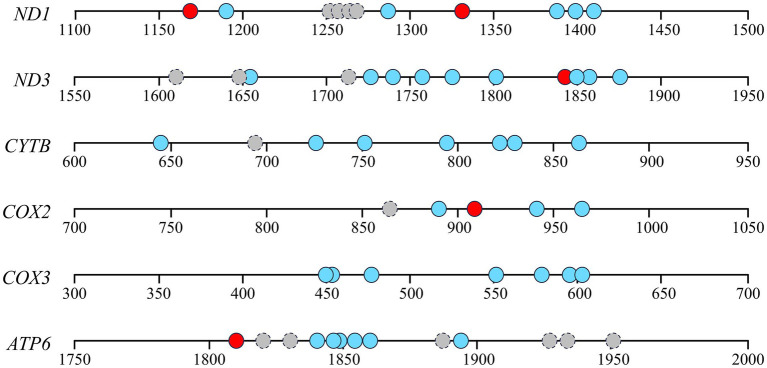
Gene structures and methylation sequencing of detected promoter regions of *ND1*, *ND3*, *CYTB*, *COX2*, *COX3* and *ATP6*. Circles mean the locations of CpG sites. Red or blue circles indicate CpG sites with significantly different or similar DNA methylation levels among the three groups. Gray circles with dotted lines indicate unanalyzable CpG sites.

**Figure 4:**
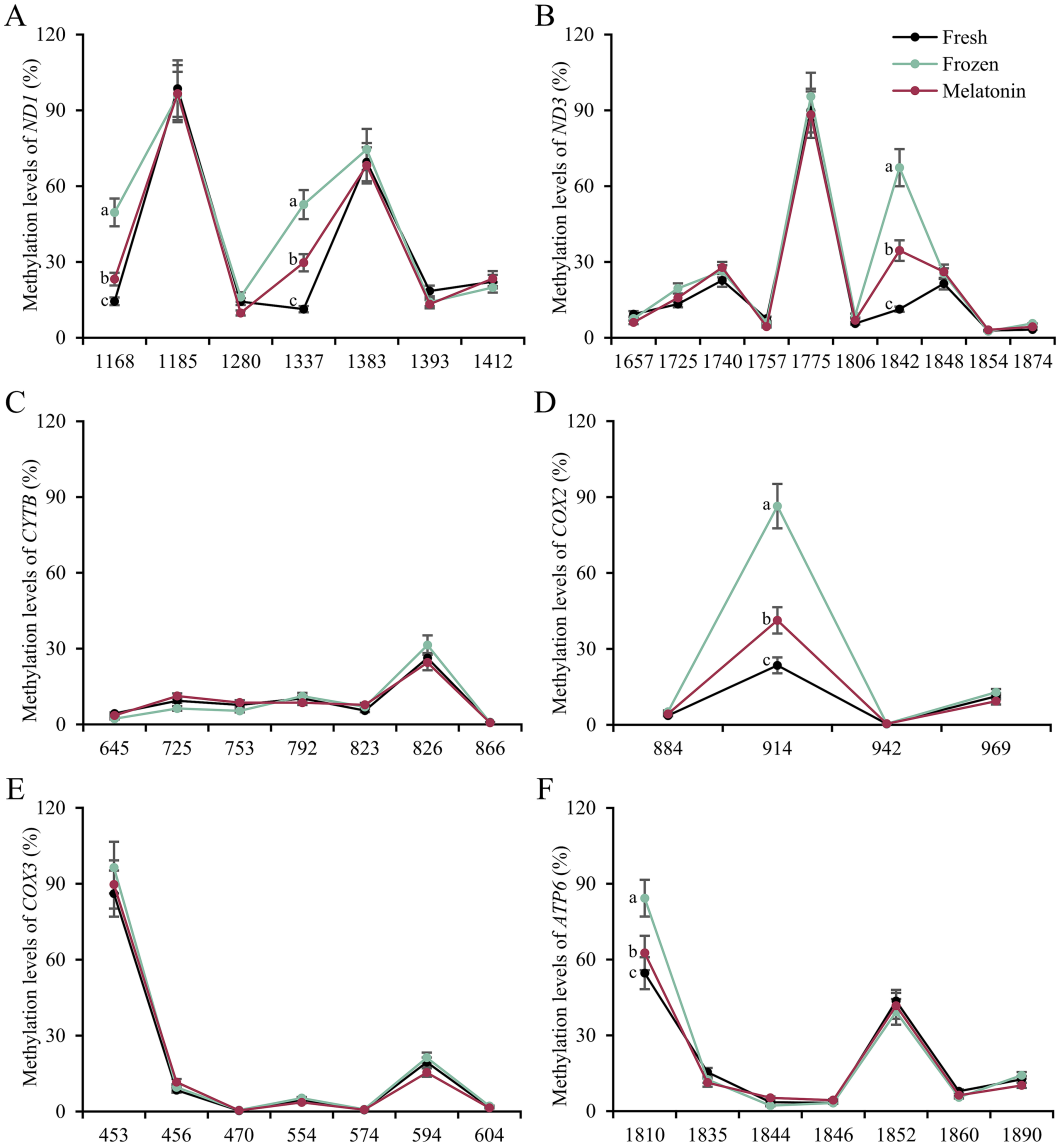
Methylation levels of detected mtDNA CpG sites in sperm from fresh, frozen and melatonin groups. The values in horizontal axis mean CpG sites of different genes including *ND1* **(A)**, *ND3* **(B)**, *CYTB* **(C)**, *COX2* **(D)**, *COX3* **(E)** and *ATP6* **(F)**. Different letters indicate the significant difference between two groups with *p* < 0.05.

**Figure 5:**
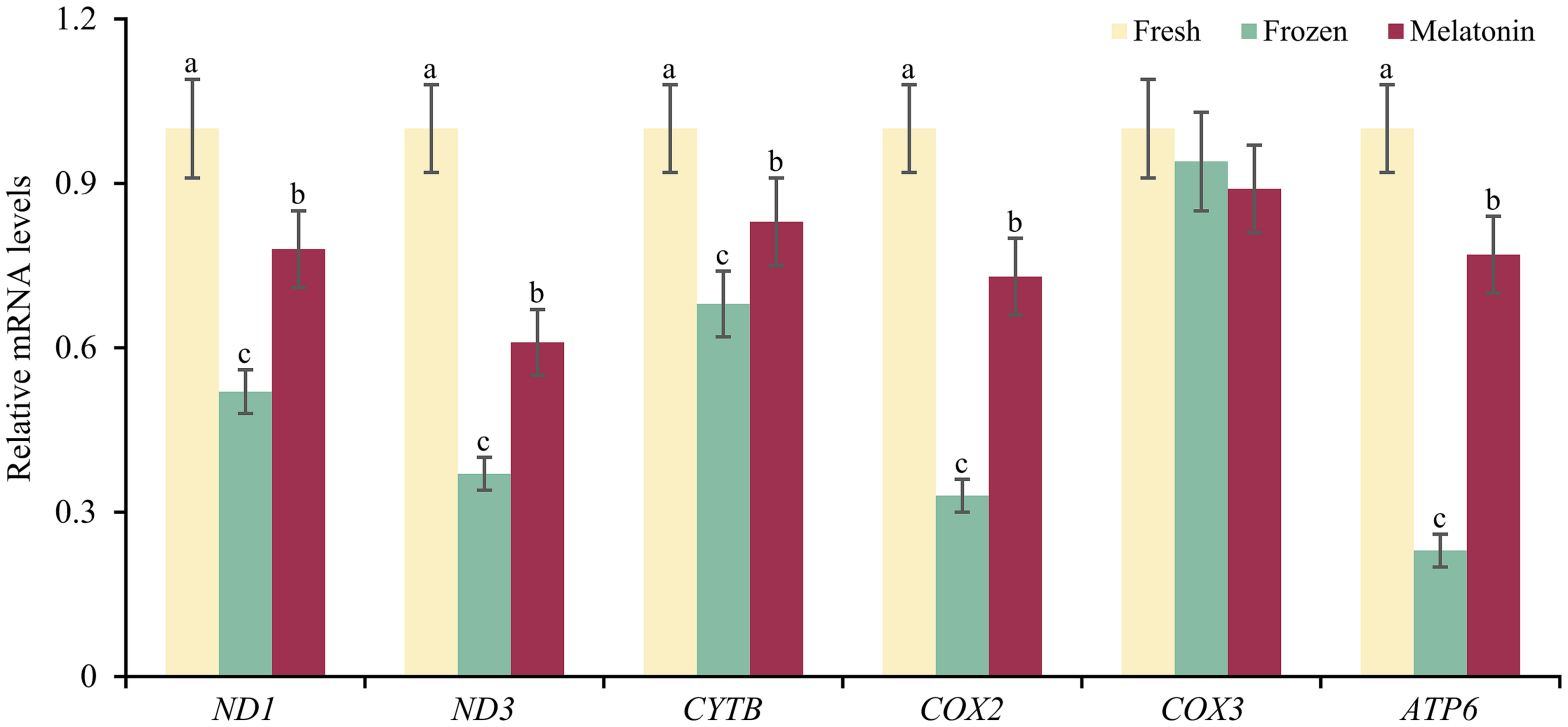
Gene expressions of mitochondria respiratory chain complex encoded genes in sperm from fresh, frozen and melatonin groups. Different letters indicate the significant difference between two groups with *p* < 0.05.

Considering the relevance between respiratory chain complex activity and related mitochondrial gene expression, we conducted gene structure and methylation sequencing analyses on the promoter regions of *ND1*, *ND3*, *CYTB*, *COX2*, *COX3* and *ATP6*. It was found that 7, 10, 7, 4, 7, 7 and 7 CpG sites were detected in the above-mentioned gene regions, respectively (*p* < 0.05, Figure 3). A total of 5 differential methylation regions were detected in 4 genes among the three groups, including two CpG sites (CpG1168 and CpG1337) of *ND1*, one CpG site (CpG1842) of *ND3*, one CpG site (CpG914) of *COX3* and one CpG site (CpG1810) of *ATP6*. Strikingly, all of 5 differential methylation regions showed a methylation level above 50% in frozen group with significant differences compared to control group, implying state transitions of gene expression from activity to inhibition. However, melatonin supplement can reduce the methylation level of above CpG sites distinctly (*p* < 0.05, Figure 4). Furthermore, mRNA abundance of these genes from quantitative real-time PCR (qRT-PCR) results supported the changes of CpG methylation. Except for *COX3*, the expression levels of *ND1*, *ND3*, *CYTB*, *COX2* and *ATP6* in melatonin group were higher than those of frozen group, but lower than those of control group (*p* < 0.05, Figure 5).

**Figure 6:**
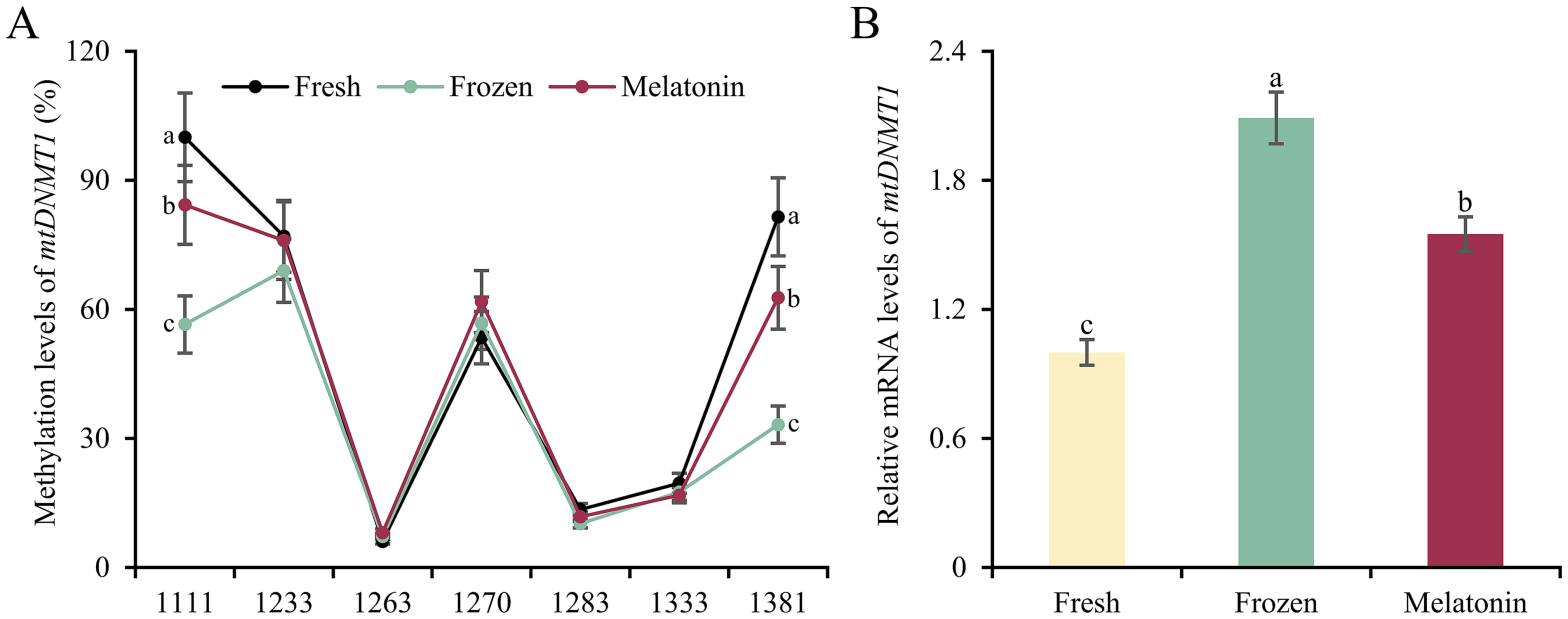
Promoter methylation **(A)** and gene expression **(B)** of *mtDNMT1* in sperm from fresh, frozen and melatonin groups. Different letters indicate the significant difference between two groups with *p* < 0.05.

Accordingly, we determined the promoter methylation level and mRNA abundance of *mtDNMT1*, the key DNA methyltransferase responsible for mtDNA methylation. Conversely, *mtDNMT1* had higher methylation level at two CpG sites in fresh group than frozen group, and melatonin supplement partially restored the low methylation state in frozen-thawed sperm (*p* < 0.05, Figure 6A). As a result, mRNA abundance of *mtDNMT1* in melatonin group was lower than frozen group, but higher than the control (*p* < 0.05, Figure 6B).

### 3.4 Effects of melatonin receptors on sperm mtDNA methylation and ATP production

**Figure 7:**
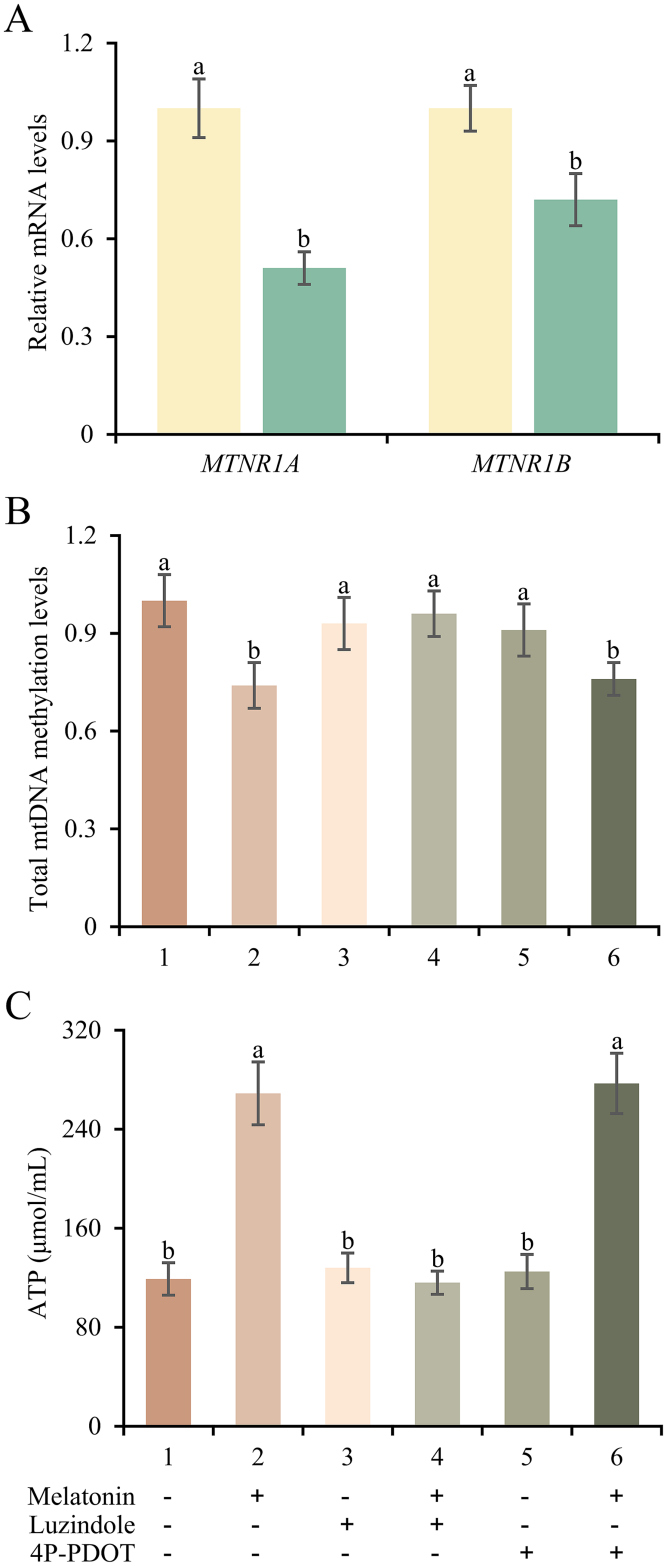
Expression changes and potential actions of melatonin receptors in frozen-thawed sperm. **(A)** mRNA expressions of *MTNR1A* and *MTNR1B* in sperm from fresh and frozen groups. **(B,C)** Total mtDNA methylation levels **(B)** and ATP concentrations **(C)** of frozen-thawed sperm treated with melatonin, luzindole or 4P-PDOT separately or jointly. Different letters indicate the significant difference between two groups with *p* < 0.05.

Next, we examined whether the protective function of melatonin on mitochondrial respiratory chain genes depended on its receptors in frozen-thawed sperm. Obviously, the expression levels of *MTNR1A* and *MTNR1B* in frozen groups were lower than those of fresh group (*p* < 0.05; Figure 7A). Furthermore, we chose two antagonists of melatonin receptors to detect the action mode of melatonin in frozen-thawed sperm. As shown in Figure 7B, total mtDNA methylation levels of frozen-thawed sperm were decreased by 26% after melatonin supplement. However, luzindole supplement blocked the effect of melatonin while 4P-PDOT has no function. The ATP content detection in frozen-thawed sperm further confirmed the effect of melatonin and its receptors on mitochondrial respiratory chain function (Figure 7C). It implies that melatonin could serve the protective function for frozen-thawed sperm through MTNRA1 rather than MTNR1B.

## 4 Discussion

Mitochondrion is the sperm substructure most sensitive to cryopreservation. Damaged spatial structure and metabolic function in mitochondria decrease the motility and fertility of frozen-thawed sperm (22, 23). Considering the low transcription activity of nuclear genome DNA in sperm, mitochondrial abnormality is closely related to the epigenetic changes of mitochondrial respiratory chain genes (24–26). Previous studies have found that the enhancing effect of melatonin on reproductive function should be relevant to the alleviative epigenetic abnormalities of mitochondria (19, 20). However, this speculation has not been verified in sperm cryopreservation (27). In this study, we proved that melatonin supplement contributed to decreased promoter methylation and enhanced gene expression of mitochondrial respiratory chain genes in frozen-thawed sperm caused by the action of mtDNMT1. Melatonin played a crucial role in sperm respiratory chain activity, oxygen utilization and ATP production through its receptor MTNR1A, which could be beneficial to the motility and fertility of frozen-thawed sperm.

To reach an oocyte and accomplish fertilization process, mammalian sperm require mitochondria to provide sufficient energy to perform capacitation, hyperactivation and acrosome reaction. Given mitochondria act as both primary synthesis site and principal target for melatonin (28), the relationship between them has been discussed extensively. Biological effects of melatonin reflect its ability to alleviate harmful reductions of membrane potential (29), improve oxidative phosphorylation (4, 30) and relieve oxidative stress (31) in mitochondria. Similarly, we found that melatonin inhibited sperm apoptosis and facilitated respiratory metabolism in frozen-thawed sperm. This could enable sperm to survive in the female reproductive tract and complete fertilization. Melatonin can localize Bcl-2 to mitochondria and inactivate Bax in mitochondria to prevent sperm apoptosis (32–34). Meanwhile, melatonin can also remove reactive oxygen and nitrogen species caused by sperm cryopreservation which would inhibit apoptosis related signaling (35). Consequently, melatonin can reduce cryopreservation-induced dysmetabolism and promote motility and fertility of frozen-thawed ram sperm.

During fertilization, the energy demands of ram sperm are mainly provided by OXPHOS pathways. OXPHOS, a major mitochondrial reaction, apparently has a more important role than glycolysis in supplying energy in ram sperm. Electron transport chain activity of sperm mitochondria is responsible for the efficiency of ATP formation, achieving energy supply through the OXPHOS pathway, which is important to sustain sperm viability and motility (36). In the presence of mitochondrial inhibitors, the reaction efficiency of OXPHOS decreases rapidly to cause a dramatic decline in sperm ATP production (35). This dependence between ROS production and fertility can be explained by the source of ROS being the mitochondrial electron transport chain, in which 1%–3% O_2_ reduction in mitochondria during superoxide formation (37). We confirmed that sperm cryopreservation caused the declined encoding gene expression and synthase activity of respiratory chain complexes, just like fish sperm (38). Moreover, the promotion of melatonin on oxidative phosphorylation has been proved in ram sperm (4). We further confirmed that melatonin supplement enhanced the OXPHOS process and ATP production to mitochondrial dysfunction. The effects of exogenous melatonin on respiratory chain complexes have also verified in numerous models of metabolic disorders (39–41).

Compromised sperm metabolism and fertilization efficiency can be attributed to abnormal mitochondrial gene expression (42). The transcriptional activity of mitochondrial genes was significantly inhibited by sperm cryopreservation, along with elevated promoter methylation. Abnormal mtDNA epigenetic modifications may also lead to changed oxidative phosphorylation activity, and in turn affect oxygen consumption, ATP and ROS production (43). On the contrary, decreased methylation caused by melatonin may be helpful to synthesize and store sufficient quantities of respiratory chain key enzymes in frozen-thawed sperm, which should be due to some receptor-mediated epigenetic mechanisms (44, 45). The epigenetic regulation of melatonin is evident as an inducer of gene expression, including abnormal expression for DNMTs and HDACs (46–48). As stated in our study, mtDNMT1 plays a key role in frozen-thawed ram sperm. It can be supported by the evidence that *mtDNMT1* overexpression results in reduced ND6 and raised ND1 in mitochondria (11). Melatonin receptors could involve this epigenetic effect, given their location in the sperm midpiece (49). Based on the different consequences of luzindole and 4P-PDOT treatment on frozen-thawed sperm, we inferred that MTNR1A, rather than MTNR1B, was responsible for the antagonistic effect of melatonin on mitochondrial gene methylation. The priority of MTNR1A is also affirmed in hamster sperm (50). This epigenetic regulation should be essential to mitochondrial protective effects in ram sperm.

In conclusion, we demonstrated that melatonin attenuated cryopreservation-induced dysmetabolism in frozen-thawed ram sperm and improved sperm motility and fertility. This protective effect of melatonin was achieved by regulating promoter methylation and expression levels of mitochondrial respiratory chain genes via MTNR1A. This study provided new perspectives on the role of epigenetic mechanisms in sperm metabolism and improved strategies of sperm cryopreservation in livestock.

## Data Availability

The original contributions presented in the study are included in the article/supplementary material, further inquiries can be directed to the corresponding authors.

## References

[1] Park YJ Pang MG. Mitochondrial functionality in male fertility: from spermatogenesis to fertilization. Antioxidants. (2021) 10:98. doi: 10.3390/antiox10010098, PMID: 33445610 PMC7826524

[2] Tiwari S Dewry RK Srivastava R Nath S Mohanty TK. Targeted antioxidant delivery modulates mitochondrial functions, ameliorates oxidative stress and preserve sperm quality during cryopreservation. Theriogenology. (2022) 179:22–31. doi: 10.1016/j.theriogenology.2021.11.013, PMID: 34823058

[3] Cordoba M Pintos LN Beconi MT. Heparin and quercitin generate differential metabolic pathways that involve aminotransferases and LDH-X dehydrogenase in cryopreserved bovine spermatozoa. Theriogenology. (2007) 67:648–54. doi: 10.1016/j.theriogenology.2006.09.041, PMID: 17084443

[4] Fang Y Zhao C Xiang H Zhao X Zhong R. Melatonin inhibits formation of mitochondrial permeability transition pores and improves oxidative phosphorylation of frozen-thawed ram sperm. Front Endocrinol. (2019) 10:896. doi: 10.3389/fendo.2019.00896, PMID: 31969863 PMC6960123

[5] Geng Q Gao R Sun Y Chen S Sun L Li W. Mitochondrial DNA content and methylation in sperm of patients with asthenozoospermia. J Assist Reprod Genet. (2024) 41:2795–805. doi: 10.1007/s10815-024-03236-0, PMID: 39190228 PMC11535106

[6] Leroux E Khorami HH Angers A Angers B Breton S. Mitochondrial epigenetics brings new perspectives on doubly uniparental inheritance in bivalves. Sci Rep. (2024) 14:31544. doi: 10.1038/s41598-024-83368-6, PMID: 39733193 PMC11682101

[7] Alipour-Jenaghard P Daghigh-Kia H Masoudi R Hatefi A. MitoQ preserves epigenetic modifications and quality parameters of rooster sperm during cryopreservation process. Reprod Domest Anim. (2025) 60:e70012. doi: 10.1111/rda.70012, PMID: 39963986

[8] Aurich C Schreiner B Ille N Alvarenga M Scarlet D. Cytosine methylation of sperm DNA in horse semen after cryopreservation. Theriogenology. (2016) 86:1347–52. doi: 10.1016/j.theriogenology.2016.04.077, PMID: 27242182

[9] Salehi M Mahdavi AH Sharafi M Shahverdi A. Cryopreservation of rooster semen: evidence for the epigenetic modifications of thawed sperm. Theriogenology. (2020) 142:15–25. doi: 10.1016/j.theriogenology.2019.09.030, PMID: 31574396

[10] Jia G Fu X Cheng K Yue M Jia B Hou Y. Spermatozoa cryopreservation alters pronuclear formation and zygotic DNA demethylation in mice. Theriogenology. (2015) 83:1000–6. doi: 10.1016/j.theriogenology.2014.11.036, PMID: 25547286

[11] Saini SK Mangalhara KC Prakasam G Bamezai RNK. DNA methyltransferase1 (DNMT1) isoform3 methylates mitochondrial genome and modulates its biology. Sci Rep. (2017) 7:1525. doi: 10.1038/s41598-017-01743-y, PMID: 28484249 PMC5431478

[12] Dou X Boyd-Kirkup JD Mcdermott J Zhang X Li F Rong B. The strand-biased mitochondrial DNA methylome and its regulation by DNMT3A. Genome Res. (2019) 29:1622–34. doi: 10.1101/gr.234021.117, PMID: 31537639 PMC6771398

[13] He C Wang J Zhang Z Yang M Li Y Tian X. Mitochondria synthesize melatonin to ameliorate its function and improve mice oocyte’s quality under *in vitro* conditions. Int J Mol Sci. (2016) 17:939. doi: 10.3390/ijms17060939, PMID: 27314334 PMC4926472

[14] Acuna-Castroviejo D Escames G Rodriguez MI Lopez LC. Melatonin role in the mitochondrial function. Front Biosci. (2007) 12:947–63. doi: 10.2741/2116, PMID: 17127351

[15] Sun TC Li HY Li XY Yu K Deng SL Tian L. Protective effects of melatonin on male fertility preservation and reproductive system. Cryobiology. (2020) 95:1–8. doi: 10.1016/j.cryobiol.2020.01.018, PMID: 32001217

[16] Shao R Wang Y He C Chen L. Melatonin and its emerging physiological role in reproduction: a review and update. Curr Mol Med. (2024) 24:449–56. doi: 10.2174/1566524023666230417103201, PMID: 37070447

[17] Najafi A Adutwum E Yari A Salehi E Mikaeili S Dashtestani F. Melatonin affects membrane integrity, intracellular reactive oxygen species, caspase3 activity and AKT phosphorylation in frozen thawed human sperm. Cell Tissue Res. (2018) 372:149–59. doi: 10.1007/s00441-017-2743-4, PMID: 29196809

[18] Casao A Mendoza N Perez-Pe R Grasa P Abecia JA Forcada F. Melatonin prevents capacitation and apoptotic-like changes of ram spermatozoa and increases fertility rate. J Pineal Res. (2010) 48:39–46. doi: 10.1111/j.1600-079X.2009.00722.x, PMID: 19919602

[19] Fang Y Deng S Zhang J Liu H Li Y Zhang X. Melatonin-mediated development of ovine cumulus cells, perhaps by regulation of DNA methylation. Molecules. (2018) 23:494. doi: 10.3390/molecules23020494, PMID: 29473888 PMC6017080

[20] Fang Y Zhang J Li Y Guo X Li J Zhong R. Melatonin-induced demethylation of antioxidant genes increases antioxidant capacity through Roralpha in cumulus cells of prepubertal lambs. Free Radic Biol Med. (2019) 131:173–83. doi: 10.1016/j.freeradbiomed.2018.11.027 30472366

[21] Fang Y Blair H Zhong RZ Zhong R Sun H Zhou D. Optimizing the freezing rate for ovine semen cryopreservation: phospholipid profiles and functions of the plasma membrane and quality and fertilization of spermatozoa. Small Rumin Res. (2016) 139:46–51. doi: 10.1016/j.smallrumres.2016.04.012

[22] Escada-Rebelo S Cristo MI Ramalho-Santos J Amaral S. Mitochondria-targeted compounds to assess and improve human sperm function. Antioxid Redox Signal. (2022) 37:451–80. doi: 10.1089/ars.2021.0238, PMID: 34847742

[23] Zhang L Sun Y Jiang C Sohail T Sun X Wang J. Damage to mitochondria during the cryopreservation, causing ROS leakage, leading to oxidative stress and decreased quality of ram sperm. Reprod Domest Anim. (2024) 59:e14737. doi: 10.1111/rda.14737, PMID: 39470252

[24] Flores E Ramio-Lluch L Bucci D Fernández-Novell JM Peña A Rodríguez-Gil JE. Freezing-thawing induces alterations in histone H1-DNA binding and the breaking of protein-DNA disulfide bonds in boar sperm. Theriogenology. (2011) 76:1450–64. doi: 10.1016/j.theriogenology.2011.05.039, PMID: 21855992

[25] Zeng C Peng W Ding L He L Zhang Y Fang D. A preliminary study on epigenetic changes during boar spermatozoa cryopreservation. Cryobiology. (2014) 69:119–27. doi: 10.1016/j.cryobiol.2014.06.003, PMID: 24974820

[26] Li Y Zhang J Shen Q Yu Z. Dual function of a fungal Gpcr in activating mitochondrial respiration and initiating prey hunting in fungus-nematode interactions. Innov Life. (2024) 2:100087. doi: 10.59717/j.xinn-life.2024.100087

[27] Korkmaz A Reiter RJ. Epigenetic regulation: a new research area for melatonin? J Pineal Res. (2008) 44:41–4. doi: 10.1111/j.1600-079X.2007.00509.x, PMID: 18078446

[28] Boutin JA Kennaway DJ Jockers R. Melatonin: facts, extrapolations and clinical trials. Biomolecules. (2023) 13:943. doi: 10.3390/biom13060943, PMID: 37371523 PMC10295901

[29] Furuta T Nakagawa I Yokoyama S Morisaki Y Saito Y Nakase H. Melatonin-induced postconditioning suppresses NMDA receptor through opening of the mitochondrial permeability transition pore via melatonin receptor in mouse neurons. Int J Mol Sci. (2022) 23:3822. doi: 10.3390/ijms23073822, PMID: 35409182 PMC8998233

[30] Khaldy H Escames G Leon J Bikjdaouene L Acuña-Castroviejo D. Synergistic effects of melatonin and deprenyl against MPTP-induced mitochondrial damage and DA depletion. Neurobiol Aging. (2003) 24:491–500. doi: 10.1016/S0197-4580(02)00133-1, PMID: 12600724

[31] Govender J Loos B Marais E Engelbrecht AM. Mitochondrial catastrophe during doxorubicin-induced cardiotoxicity: a review of the protective role of melatonin. J Pineal Res. (2014) 57:367–80. doi: 10.1111/jpi.12176, PMID: 25230823

[32] Li W Fan M Chen Y Zhao Q Song C Yan Y. Melatonin induces cell apoptosis in AGS cells through the activation of JNK and P38 MAPK and the suppression of nuclear factor-kappa B: a novel therapeutic implication for gastric cancer. Cell Physiol Biochem. (2015) 37:2323–38. doi: 10.1159/000438587, PMID: 26645893

[33] Yang Y Jiang S Dong Y Fan C Zhao L Yang X. Melatonin prevents cell death and mitochondrial dysfunction via a SIRT1-dependent mechanism during ischemic-stroke in mice. J Pineal Res. (2015) 58:61–70. doi: 10.1111/jpi.12193, PMID: 25401748

[34] Li Q Chen S Yin Y Han M Shi H Gong Y. Intracellular and intercellular crosstalk between exosomes and autophagy. Innov Life. (2025) 3:100132. doi: 10.59717/j.xinn-life.2025.100132

[35] Windsor DP. Mitochondrial function and ram sperm fertility. Reprod Fertil Dev. (1997) 9:279–84. doi: 10.1071/r96109, PMID: 9261876

[36] Blanco-Prieto O Mislei B Martinez-Pastor F Spinaci M Mari G Bucci D. Study of mitochondrial function in thawed bull spermatozoa using selective electron transfer chain inhibitors. Theriogenology. (2023) 208:8–14. doi: 10.1016/j.theriogenology.2023.05.021, PMID: 37290146

[37] Desai N Sabanegh E Jr Kim T Agarwal A. Free radical theory of aging: implications in male infertility. Urology. (2010) 75:14–9. doi: 10.1016/j.urology.2009.05.025, PMID: 19616285

[38] Figueroa E Valdebenito I Zepeda AB Figueroa CA Dumorné K Castillo RL. Effects of cryopreservation on mitochondria of fish spermatozoa. Rev Aquac. (2017) 9:76–87. doi: 10.1111/raq.12105

[39] Andrabi SA Sayeed I Siemen D Wolf G Horn TFW. Direct inhibition of the mitochondrial permeability transition pore: a possible mechanism responsible for anti-apoptotic effects of melatonin. FASEB J. (2004) 18:869–71. doi: 10.1096/fj.03-1031fje, PMID: 15033929

[40] Solis-Munoz P Solis-Herruzo JA Fernandez-Moreira D Gómez-Izquierdo E García-Consuegra I Muñoz-Yagüe T. Melatonin improves mitochondrial respiratory chain activity and liver morphology in ob/ob mice. J Pineal Res. (2011) 51:113–23. doi: 10.1111/j.1600-079X.2011.00868.x, PMID: 21355880

[41] Teodoro BG Baraldi FG Sampaio IH Bomfim LHM Queiroz AL Passos MA. Melatonin prevents mitochondrial dysfunction and insulin resistance in rat skeletal muscle. J Pineal Res. (2014) 57:155–67. doi: 10.1111/jpi.12157, PMID: 24981026

[42] Wai T Ao A Zhang X Cyr D Dufort D Shoubridge EA. The role of mitochondrial DNA copy number in mammalian fertility. Biol Reprod. (2010) 83:52–62. doi: 10.1095/biolreprod.109.080887, PMID: 20130269 PMC2888963

[43] Sharma N Pasala MS Prakash A. Mitochondrial DNA: epigenetics and environment. Environ Mol Mutagen. (2019) 60:668–82. doi: 10.1002/em.22319, PMID: 31335990 PMC6941438

[44] Wang F Tian X Zhang L Gao C He C Fu Y. Beneficial effects of melatonin on in vitro bovine embryonic development are mediated by melatonin receptor 1. J Pineal Res. (2014) 56:333–42. doi: 10.1111/jpi.12126, PMID: 24666110

[45] Cui P Yu M Luo Z Dai M Han J Xiu R. Intracellular signaling pathways involved in cell growth inhibition of human umbilical vein endothelial cells by melatonin. J Pineal Res. (2008) 44:107–14. doi: 10.1111/j.1600-079X.2007.00496.x, PMID: 18078456

[46] Tain YL Huang LT Chan JY. Transcriptional regulation of programmed hypertension by melatonin: an epigenetic perspective. Int J Mol Sci. (2014) 15:18484–95. doi: 10.3390/ijms151018484, PMID: 25318052 PMC4227227

[47] Tian X Wang F He C Zhang L Tan D Reiter RJ. Beneficial effects of melatonin on bovine oocytes maturation: a mechanistic approach. J Pineal Res. (2014) 57:239–47. doi: 10.1111/jpi.12163, PMID: 25070516

[48] Sharma R Ottenhof T Rzeczkowska PA Niles LP. Epigenetic targets for melatonin: induction of histone H3 hyperacetylation and gene expression in C17.2 neural stem cells. J Pineal Res. (2008) 45:277–84. doi: 10.1111/j.1600-079X.2008.00587.x, PMID: 18373554

[49] Talpur HS Worku T Rehman ZU Dad R Bhattarai D Bano I. Knockdown of melatonin receptor 1 and induction of follicle-stimulating hormone on the regulation of mouse granulosa cell function. Reprod Biol. (2017) 17:380–8. doi: 10.1016/j.repbio.2017.10.005 29097083

[50] Weaver DR Liu C Reppert SM. Nature’s knockout: the Mel1b receptor is not necessary for reproductive and circadian responses to melatonin in Siberian hamsters. Mol Endocrinol. (1996) 10:1478–87. doi: 10.1210/mend.10.11.8923472, PMID: 8923472

